# Current progress and open challenges for applying deep learning across the biosciences

**DOI:** 10.1038/s41467-022-29268-7

**Published:** 2022-04-01

**Authors:** Nicolae Sapoval, Amirali Aghazadeh, Michael G. Nute, Dinler A. Antunes, Advait Balaji, Richard Baraniuk, C. J. Barberan, Ruth Dannenfelser, Chen Dun, Mohammadamin Edrisi, R. A. Leo Elworth, Bryce Kille, Anastasios Kyrillidis, Luay Nakhleh, Cameron R. Wolfe, Zhi Yan, Vicky Yao, Todd J. Treangen

**Affiliations:** 1 Department of Computer Science, Rice University, Houston, TX USA; 2 Department of Electrical Engineering and Computer Sciences, University of California Berkeley, Berkeley, CA USA; 3 Department of Biology and Biochemistry, University of Houston, Houston, TX USA; 4 Department of Electrical and Computer Engineering, Rice University, Houston, TX USA; 5 Department of Bioengineering, Rice University, Houston, TX USA

**Keywords:** Computer science, Computational biology and bioinformatics, Machine learning

## Abstract

Deep Learning (DL) has recently enabled unprecedented advances in one of the grand challenges in computational biology: the half-century-old problem of protein structure prediction. In this paper we discuss recent advances, limitations, and future perspectives of DL on five broad areas: protein structure prediction, protein function prediction, genome engineering, systems biology and data integration, and phylogenetic inference. We discuss each application area and cover the main bottlenecks of DL approaches, such as training data, problem scope, and the ability to leverage existing DL architectures in new contexts. To conclude, we provide a summary of the subject-specific and general challenges for DL across the biosciences.

## Abstract (web summary)

Deep learning has enabled advances in understanding biology. In this review, the authors outline advances, and limitations of deep learning in five broad areas and the future challenges for the biosciences.

## Introduction

**Fig. 1:**
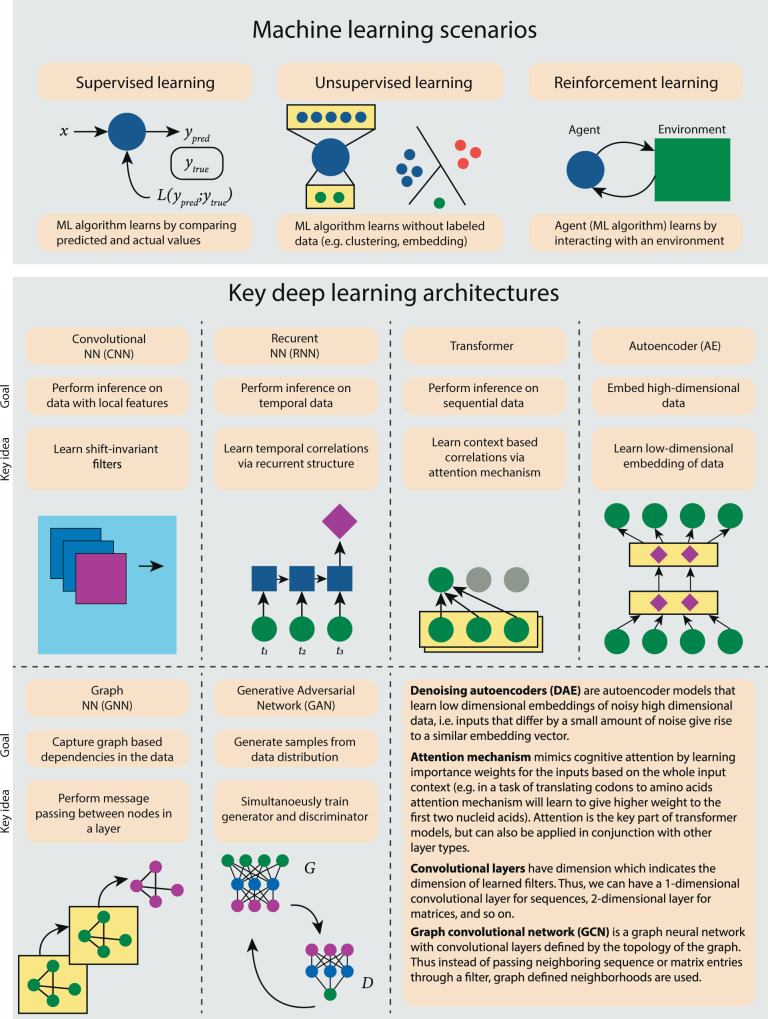
An overview of machine learning scenarios and commonly used DL architectures. Top panel encapsulates the three most common paradigms of machine learning: supervised learning in which dataset contains ground truth labels, unsupervised learning in which dataset does not contain ground truth labels, and reinforcement learning in which an algorithmic agent interacts with a real or simulated environment. The bottom panels provide an overview of the most prevalent DL architecture ideas each designed to achieve specific highlighted goals. An additional set of short descriptions is provided for other common components of DL architectures mentioned in the manuscript.

The recent success of AlphaFold2^1^ in predicting the 3D structure of proteins from their sequences highlights one of the most effective applications of deep learning in computational biology to date. Deep learning (DL) allows for finding a representation of the data with multiple layers of abstraction using complex models that are composed of several layers of nonlinear computational units (Fig. 1). Observed through the success of DL in a broad variety of application domains, the efficacy of using DL depends on the development of specialized neural network architectures that can capture important properties of the data such as spatial locality (convolutional neural networks – CNNs), sequential nature (recurrent neural networks – RNNs), context dependence (Transformers), and data distribution (autoencoders – AEs). Figure 1 illustrates six DL architectures that have found the most applications within the realm of computational biology. We refer the reader to LeCun et al. ^2^ for a complete review of DL methods and architectures and keep the focus of the paper on computational biology applications. These DL models have revolutionized speech recognition, visual object recognition, and object detection and have lately played a key role in solving important problems in computational biology. The applications of DL in other areas of computational biology, such as functional biology, are only growing while other areas, such as phylogenetics, are in their infancy. Given the wide divide between the receptiveness of DL in different areas in computational biology, some key questions remain unanswered: (1) What makes an area prime for DL methods? (2) What are the potential limitations of DL for computational biology applications? (3) Which DL model is most appropriate for a specific application area in computational biology?

**Fig. 2:**
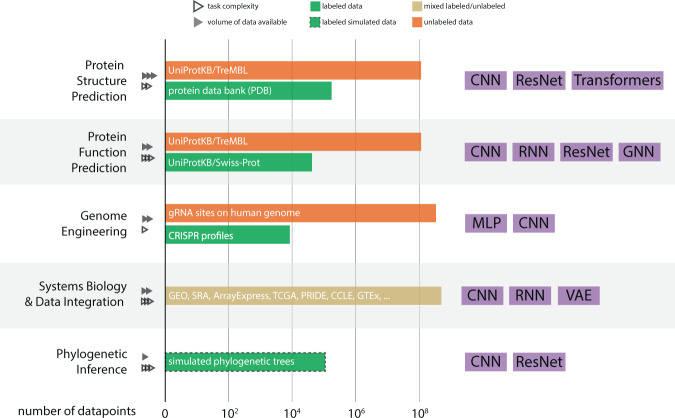
A summary view of the major labeled and unlabeled datasets, and the architectures being used in deep-learning methods in computational biology. For each of the areas considered in this manuscript, it summarizes estimated sizes of key datasets and databases, as well as the projected growth rate of these. Additionally the rightmost column summarizes the most popular DL architectures applied to the corresponding areas in biosciences.

In this paper, we aim to address these foundational questions from the lens of computational biology. The answers, however, are highly task specific and can only be addressed in the context of the corresponding applications. The pitfalls of applying machine learning (ML) in genomics have been discussed in Whalen et al.^3^, but our goal is to provide a perspective on the impact of DL across five distinct areas. While there are multiple areas of interest in the biosciences where DL has achieved notable successes (e.g. DeepVariant^4^, DeepArg^5^, metagenomic binning^6^, and lab-of-origin attribution^7^), we aim to only focus on a few diverse and broad subtopics. In those areas we evaluate the improvements that DL has had over classical ML techniques in computational biology with varying levels of success to date (Fig. 2). For each area, we explore limitations of current approaches and opportunities for improvement, and include practical tips. We anchor our discussions around five broad, distinct areas in computational biology: protein structure prediction, protein function prediction, genome engineering, systems biology and data integration, and phylogenetic inference (Table 1). These areas provide a range of impact levels from major paradigm shifts (AlphaFold2) to DL applications in their infancy (phylogenetic inference); and collectively, they provide rich enough technical diversity to address the questions raised in this perspective. Over the next several subsections, we will review progress in each of the four computational biology topics, ordered from (i) paradigm shifting (where DL clearly outperforms other ML and classical approaches, and provides a field-wide impact), (ii) major success (where DL performance is typically higher than of that of other  ML and classical approaches), (iii) moderate success (where DL performance is typically comparable to other ML and classical approaches) to (iv) minor successes (where DL methods are not widely adopted or underperform compared to other ML and classical approaches), and then discuss common challenges for DL in biosciences (Table 2).

**Table 1:**
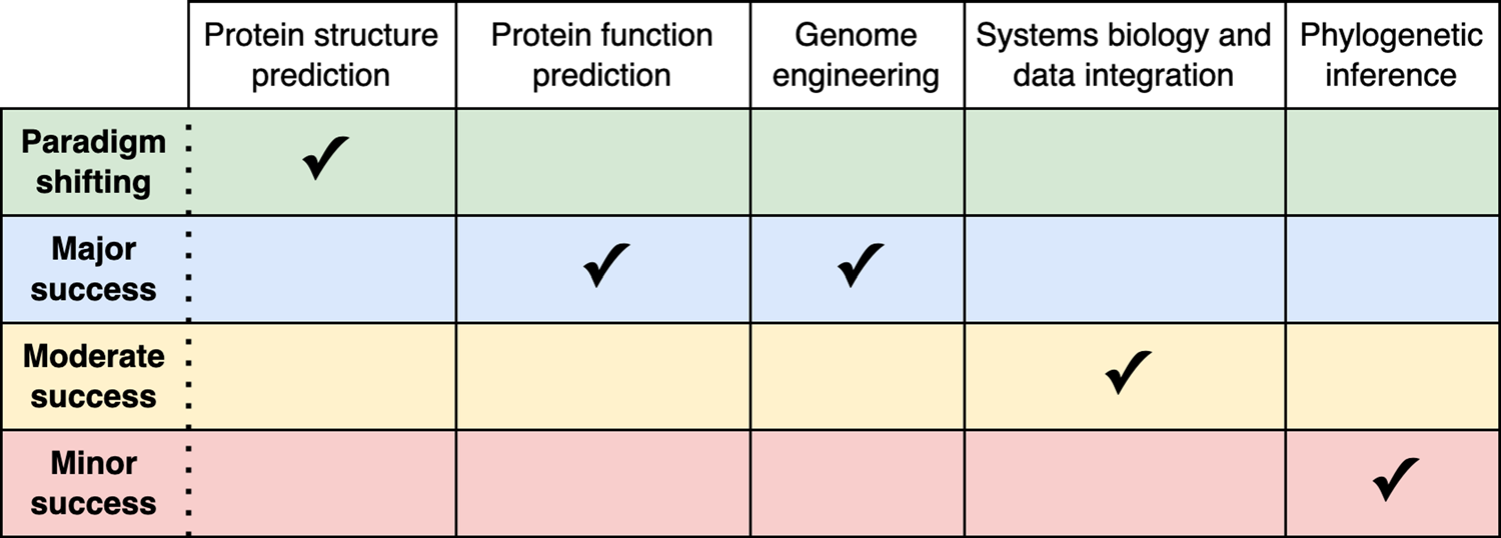
Impact of Deep Learning on Computational Biology.

Each of the subareas in biosciences considered in this manuscript is assigned a level of success of the DL applications based on the relative performance of DL as compared to other ML and classical methods.

**Table 2:** Commonly faced challenges in computational biology and potential solution avenues when using DL.

Challenge	Experimental/non-DL solution	DL solution
Biased results	Improve study design	Identify forms and sources of technical bias
		Fair AI approaches
High infrastructure costs	Optimize code performance	Optimize DL architecture
	Parallelize code	Parallelize to low-cost devices
	Sub-sample analyzed data	Condense training data (e.g. coresets)
Lack of explainability	Statistical analyses	Explainable post-hoc methods
Limited training data	Generate and label more data	Data augmentation (e.g. GANs)
Overfitting	Regularization	Dropout
		Early stopping
		Smaller models
		Additional training data
Poor performance on novel data	Expand databases	Use larger models
		Analyze generalization potential

## Paradigm shifting successes of DL

### Protein structure prediction

We start our discussion with protein structure prediction which is arguably one of the most successful applications of DL in computational biology; this success is what we refer to as a paradigm shift. It is largely known that the protein’s amino acid sequence determines its 3D structure, which is in turn directly related to its function (e.g., chemical reaction catalysis, signal transduction, scaffold, etc.)^8,9^. The history of protein structure prediction problem goes back to the determination of the 3D structure of myoglobin by John Kendrew in the 1950s which was a landmark in biochemistry and structural biology^10^. Since then, X-ray crystallography has become the gold-standard experimental method for protein structure determination^11,12^, as well as the reference to validate computational models for protein structure prediction. Considering the high cost and technical limitations of X-ray crystallography, and the growing access to biological sequences following the Human Genome Project, predicting the 3D structure of a protein from its sequence became the Mount Everest in computational biology^8^; a challenge broadly known as the “protein folding problem”. Initial efforts concentrated on the use of biophysically-accurate energy functions and knowledge-based statistical reasoning, but faster progress was recently achieved with a greater focus on DL.

One of the key reasons for the recent success of DL in this area has been the wealth of unsupervised data in the form of multiple sequence alignment (MSA)^1,9,13–17^, which has enabled learning a nonlinear evolution-informed representation of proteins. Progress in the field has been accelerated by the creation of a bi-annual international competition, called the Critical Assessment of Protein Structure Prediction (CASP). Launched in 1994, CASP created the means to objectively test available methods through blind predictions, providing competing groups with a set of challenges (i.e., sequences of proteins with unknown structures), and evaluating their performances against the respective experimentally-determined structures. In their first participation in CASP13, AlphaFold, implemented by DeepMind group at Google, made the news by clearly outperforming the second best method^14^, and nearly twice beyond the projection based on previous editions^18^. Following recent trends in the field^13,16,19,20^, AlphaFold and AlphaFold2 leverage the combined use of DL and MSA^18,21^. This proved to be a winning strategy which was able to overcome the lack of large training datasets on protein structure. The Protein Data Bank (PDB)^22^ is the reference database for experimentally-determined macromolecular structures, and currently hosts close to 180,000 entries. This is a small number of data points for a complex mapping involved in the problem, and these are further biased by technical constraints of the experimental methods. Protein sequence data, on the other hand, is available on a much larger scale. Therefore, MSA allows modeling methods to extract pairwise evolutionary correlations from this larger corpus of data, maximizing the learning on available structural data. Other key factors for the success of DL in this area include innovation in model design such as new attention strategies tuned towards invariances and symmetries in proteins, graph-based representations, and model recycling strategies.

The impact of AlphaFold2 on the field of structural biology is undeniable; it successfully demonstrated the use of a DL-based implementation for high accuracy protein structure prediction^21^. This achievement is already driving and accelerating further developments in the field, as highlighted by the remarkable number of early citations. In addition, DeepMind has partnered with the European Molecular Biology Laboratory (EMBL)^23^ to create an open-access database of protein structures modeled with AlphaFold2^17^. The database already covers 98.5% of human proteins, for which at least 36% of the amino acid residues were predicted with high confidence. Finally, rather than retiring experimental methods, DL-based methods might augment the accuracy and reach of experimental methods as demonstrated by preliminary applications to solving challenging structures with data from X-ray crystallography and cryo-EM^1,15^. However, many caveats, limitations and open questions^8,9^ remain. In particular, while AlphaFold2 successfully predicts the static structure of a protein, many key insights about protein’s biological function come from its dynamic conformations. Furthermore, dynamics of interaction of multiple proteins still present open challenges in the field. Moving forward, it will be important to monitor the application of DL to these follow up research areas.

## Major successes of DL

### Protein function prediction

Predicting protein function is a natural next step after protein structure prediction. Protein function prediction involves mapping target proteins to curated ontologies, such as Gene Ontology (GO) terms, Biological Processes (BP), Molecular Functions (MF) and Cellular components (CC). Protein structure can convey a lot of information about these ontologies, however, there is no direct mapping between the two and the mapping is often very complex^24^. Despite the tremendous growth of protein sequences available in the UniProtKB database, functional annotations for the vast majority of proteins still remain partly or completely unknown^25^. Limited and imbalanced training examples, a large output space of possible functions and the hierarchical nature of the GO labels are some of the main bottlenecks associated with functional annotation of proteins^26^. To overcome some of the issues recent methods have leveraged features from different sources including sequence^27^, structure^22^, interaction networks^28^, scientific literature, homologies, domain information^29^ and even incorporate one or a combination of DL architectures to handle different stages of prediction task (e.g. feature representation, feature selection, and classification).

One of the most successful DL approaches to the problem, DeepGO^30^ incorporated CNN to learn sequence-level embeddings and combines it with knowledge graph embeddings for each protein obtained^31^ from Protein-Protein Interaction (PPI) networks. DeepGO was one of the first DL based models to perform better than BLAST^32^ and previous methods on functional annotation tasks over the three GO categories^30^. An improved version of the tool, DeepGOPlus^33^ emerged as one of the top performers when compared to other tools in the CAFA3 challenge across the three GO categories^33^. DeepGOPlus used convolutional filters of different sizes with individual max-pooling to learn dense feature representations of protein sequences embedded in a one-hot encoding scheme. The authors showed that combining the outputs from CNN with homology-based predictions from DIAMOND^34^ can result in better predictive accuracy.

Unsupervised methods such as DAEs also have been instrumental by learning dense, robust, and low-dimensional representations of proteins. Chicco et al.^35^ developed a DAE to represent proteins for assigning missing GO annotations and showed 6% to 36% improvements compared to non-DL methods over six different GO datasets. Miranda and Hu^36^ introduced the Stacked Denoising Autoencoders (sdAE) to learn more robust representation of proteins. Gilgorijevic et al. introduced deepNF^37^ that uses Multimodal DAE (MDA) to extract features from multiple heterogeneous interaction networks which outperform methods based on matrix factorization and linear regression ^37^. Methods for learning low-dimensional embeddings of proteins continue to grow.

Beyond just predicting Gene Ontology labels, studies have also focused on several other task-specific functional categories such as identifying specific enzyme functions^38^ and potential post-translational modification sites^39^. These studies are a fundamental step towards developing novel proteins with specialized functions or modifying the efficacy of existing proteins as seen in the recent advances of DL in enzyme engineering^40^. Going forward, applications of deep learning in engineering proteins tailored to specific functions can help increase throughput of candidate proteins for pharmaceutical applications among other domains.

Besides these canonical architectures, there have been other approaches that have used a combination of the above methods for function classification^41^. Overall, previous results indicate that models integrating features from multi-modal data types (e.g., sequence, structure, PPI, etc) are more likely to outperform the ones that rely on a single datatype. Trends from literature indicate that relying on task-specific architectures could help greatly enhance the feature representation from respective data types. Future work in this direction could focus on combining DAEs and RNNs for sequence based representation, and Graph Convolutional Networks (GCNs) for structure based as well as PPI based information. Combining these representations in a hierarchical classifier such as the multi-task DNN with biologically-relevant regularization methods^42,43^ could allow for an explainable and computationally feasible DL architecture for protein function prediction.

### Genome engineering

Biomedical engineering, and in particular genome engineering, is an important area in biology where DL models have been increasingly employed. Among genome engineering technologies, clustered regularly interspaced short palindromic repeats (CRISPR), i.e., a family of DNA sequences found in the genomes of prokaryotic organisms, have been recently used as a guide to recognize and cleave specific locations on the human genome. In the CRISPR-associated protein 9 (Cas9) technology, a single-guide RNA (gRNA) steers the protein to a specific genomic target. When the 20-nucleotide gRNA sequence complements the genome, Cas9 creates a double-strand break (DSB) on the targets (an on-target event). Due to the ability to precisely target specific locations on the genome, we have observed enormous advancements in CRISPR-based editing technologies since the development of Cas9. However, recent studies have shown that multiple mismatches between the gRNA and the genomic targets are tolerated and, as a result, Cas9 can cut unwanted locations on the genome (an off-target event). Off-target edits have pathogenic effects on the functionality and integrity of the cell. Therefore, the full clinical deployment of Cas9 has been slow due to the insufficient efficiency, reliability, and controllability challenges for therapeutic purposes. As a result, reducing off-target while improving the on-target efficiency has been an important ultimate goal in genome engineering target by DL techniques.

The sheer complexity of the biological process involved in modeling the DNA repair process and the growing availability of labeled data caused by a rapid drop in the cost of CRISPR assays, have made DL-based methods particularly successful choices to find the root cause of these inefficiencies. The use of DL models was triggered by the observation that the on-target and off-target events and the DNA repair outcome^44^ are predictable by the sequence around the DSB, its location on the genome, and the potential mistargeted sequences on the genome. Several computational tools have been successfully developed to design gRNAs with maximum on-target activity and minimum off-target effects^45^. DeepCas9 is among CNN-based models which learns functional gRNAs directly from their canonical sequence representation^46,47^. The success of DeepCRISPR, on the other hand, relies on extracting about half a billion unlabeled gRNA sequences from the human coding and non-coding regions and learning a low-dimensional representation of the gRNA^48^. DeepCRISPR also uses a data augmentation method to create less than a million sgRNAs with known knockout efficiencies to train a larger CNN model. CnnCrispr uses a language processing model to learn the representation of gRNA and then employs a combination of bidirectional LSTM and CNN^49^ while RNNs have been the reason for the success of other models^50^. Attention mechanism has also been shown to improve the accuracy in predicting on and off target effects^51,52^. ADAPT^53^ is another recent CNN-based method for fully-automated CRISPR design for vertebrate-infecting viral diagnostics which owes its success to the construction of a massive training CRISPR dataset. Recent methods for predicting the DNA repair outcome employ other strategies. SPROUT compensates the lack of labels on harder-to-collect human CD4+ T cells by predicting a summary statistics of the DNA repair outcome^54^. FORECasT employs a larger dataset from easier-to-collect human chronic myelogenous leukemia cell-line (K562)^55^. InDelphi creates hand-designed features of the input sequence including the length and GC content of the homologous sequences around the cut site^56^ while CROTON avoids feature engineering and instead performs neural architecture search^57^. All these strategies help reducing the number of labeled data points required to learn the input-output mapping.

The future of DL is geared towards new editing technologies such as CRISPR-Cas12a (cpf1)^58^, base editors^59^, and prime editors^60^. While these methods do not introduce DSBs, their efficiency is still improving^61^; in fact, DL has already shown promise in predicting the efficiency of Adenine base editors (ABEs) and Cytosine base editors (CBEs)^59^ as well as prime editor 2 (PE2) activities in human cells^60^. The future challenges, however, are in understanding these models. CRISPRLand is a recent framework which takes the first step towards interpretation and visualization of DL models in terms of higher-order interactions^62^. Besides explainablity, we speculate that methods that enable an uncertainty estimate of the prediction outcome become more prevalent in genome editing. Further, due to the significant cell-type effects on the efficiency of the CRISPR experiments, it is critical to be aware of the distribution shifts in deploying DL models in genome engineering. The integration of domain adaptation^63^ methods to limit the effect of such distribution shifts are among other important future directions.

## Moderate successes of DL

### Systems biology and data integration

Systems biology takes a holistic view of modeling complex biological processes to ultimately unravel the link between genotype and phenotype. Integration of diverse -omics data is central in bridging this gap, enabling robust predictive models that have led to several recent breakthroughs, spanning from basic biology^64^ to precision medicine. These data are now more accessible than ever, due to improvements in sequencing technologies and the establishment of open access public repositories where researchers can deposit their own studies, such as SRA^65^, GEO^65^, ArrayExpress^66^, and PRIDE^67^; and large coordinated efforts with structured multi-omic datasets: TCGA^68^, CCLE^69^, GTEx^70^, and ENCODE^71^. Given recent successes and the prevalence of both single and co-assay data, the field is now focused on integrating different data types (e.g., genomics, transcriptomics, epigenomics, proteomics, metabolomics) on single individuals, across many individuals, within and between phenotypic groups, and across different organisms. Data integration tasks fall into two main categories: 1) integration across different platforms and studies of a single data type, at times with other non-omics data (e.g., protein-protein interactions, pathway annotations, motif presence) and 2) integration between different -omic data types (e.g., RNA-seq, ChIP-seq, ATAC-seq, BS-seq). Much progress has been made on integration within a single data type, especially transcriptomics data, with classical ML and statistical approaches developed for batch correction^72–75^, modeling global gene co-expression patterns^76^, Bayesian integration strategies for function prediction^77,78^, and phenotype classification^79^. More recently, the increasing prevalence of single-cell transcriptomics has given rise to a new host of classic ML^80–82^ and DL^83,84^ approaches for data integration across experiments. DL methods in this space have arisen out of the need for methods that scale well with the large number of cells and ability to model non-linear patterns of cell similarity^83,85^. Here, we have only skimmed the surface of methods being developed for expression data, but this trend is emerging for other -omics data types, similarly driven by the resolution of improved high-resolution experimental assays^86,87^. Broadly, data integration analyses that simultaneously combine data types together, either from different studies or different types, typically fall into one of three categories, given the stage at which the integration is performed^88^: concatenation-based, transformation-based, or model-based. While data integration across studies can be data of the same type, here we focus on methods that specifically integrate across different -omics types, as these questions introduce additional technical challenges and complexity.

Concatenation-based integration methods perform data integration early in the method pipeline by combining data, in raw or processed forms, before any joint modeling and analysis. Traditional ML concatenation-based methods are often unsupervised and typically use automatic feature extraction techniques such as lasso^89^, joint clustering schemes^90^, and dimensionality reduction^91^ to find relevant signal. These methods are usually applied to well-curated, multi-omic datasets from large consortia (e.g., TCGA), and thus most often have been used to find meaningful patient subgroups characterized by distinct patterns across data modalities. More recently, autoencoders have been used as an initial data processing step to generate lower dimensional embeddings that are then concatenated together as features for downstream models^92,93^. These approaches have improved performance over existing methods likely due to the advantages autoencoders have in denoising tasks, as well as their abilities to model nonlinear latent structure, even without sample labels.

Instead of directly concatenating separate latent embeddings, some groups have pursued transformation-based integration methods by modeling data jointly by mapping to a common representation (e.g., graph or kernel matrix). Historically, classic transformation-based ML methods use known anchor references^94^, kernel^95^, or manifold methods^96^ to align multi-omics data. This is a rapidly growing area in data integration, especially for DL methods. Building off of the use of anchors from classical ML methods, new state-of-the-art methods frequently train single modality autoencoders, followed by an alignment procedure across modalities^97^. This direction is exciting, because once trained, the models can be used to predict an unobserved modality given a single data type. Additional exciting developments harness the power of these embedding representations together with other DL methods, including CNNs and RNNs for wide ranging predictive tasks, including cell fate^98^, drug response^99^, survival^92,100^, and clinical disease features^101^.

Perhaps the most straightforward way to integrate multi-modal data is to train individual data modality models, then integrate them by combining the results from the individual models, termed model-based integration. To some degree, this is similar to ensemble approaches frequently used in classical ML. Methods in this space can take wide-ranging approaches, including building data modality-specific networks before fusing them using message-passing theory^102^ or combining different data representations using a discriminative learning approach^103^. DL methods have yet to gain much momentum for model-based integration, likely because the very nature of most DL methods blurs the line between the transformation-based and model-based paradigms. Classical approaches here try to bridge data modalities by finding a common modeling space, while DL naturally can identify common representations and model them jointly, thus circumventing the need for separate modeling and integration steps. While it is clear that deep neural networks will likely lead to better performance in data integration tasks, it is also important to keep in mind the limitations of DL, as well as important areas for continued research. Specifically, it is known that DL has the tendency to overfit to data. On the other hand, in data integration tasks, batch effects can be prevalent and it is often easy to have “contamination” between the training and test sets, all of which can lead to inflated performance estimates. Thus, it is important to carefully set up truly independent evaluation sets and identify appropriate performance baselines^3^. Furthermore, while genome-wide and whole transcriptomics datasets have broad coverage across the genome and transcriptome, human data (and in some cases, model organism data) is often skewed towards a disproportional amount of sick individuals^104^, is sex-biased towards men^105^, and biased by race with an over-represented population of Europeans^106^. These biases can result in spurious associations that plague all ML methods, but may be particularly difficult to identify when using DL.

## Minor successes of DL

### Phylogenetics

**Fig. 3:**
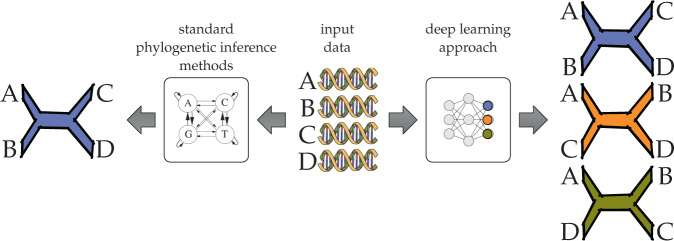
Standard and DL approaches to phylogenetic inference. The input consists of sequences (DNA sequences in this illustration) obtained from the taxa of interest. Here, the taxa are **A, B**, **C**, and **D**. In standard approaches, such as maximum likelihood and maximum parsimony, a generative model in the form of a tree whose leaves are labeled by the four taxa is inferred. In the recently introduced DL approach to phylogenetic inference, the problem is viewed as a classification task where the network outputs correspond to the three possible tree topologies whose leaves are labeled by the taxa **A**, **B**, **C**, and **D**.

A phylogeny is an evolutionary tree that models the evolutionary history of a set of taxa. The phylogeny inference problem concerns building a phylogeny from data—often molecular sequences—obtained from the set of taxa under investigation ^107^. Figure 3 illustrates the phylogeny inference problem on four taxa; in this case it can be viewed as a classification problem among three possible topologies. However, classification methods have a major limitation in that they cannot infer branch lengths, nor do they scale beyond a very small number of taxa because the number of possible topologies (classes) grows super-exponentially with this variable. But perhaps more importantly, classifiers like DL models require training data, and benchmark data where the true phylogeny is known is almost impossible to obtain in this field. Instead, simulations have been the method of choice for generating training data, but this is a major dependency and methods are known to have divergent performance on simulated and biological data^108^. For complex versions of the phylogeny inference problem, more realistic simulation protocols are needed. Finally, phylogenetic inference on a single gene is in one sense a simplified problem itself: inferring a single phylogeny from genome-wide data introduces the complication that different genes can have different histories, or the true phylogeny might be a network^109^, rather than a tree. For these reasons DL has either had limited success or been restricted to small sub-problems aside from the main inference task.

Nonetheless, there have been attempts to use DL for the classification task as described above. The Self-Organizing Tree (SOTA) algorithm^110^ is a two-decades old unsupervised hierarchical clustering method based on a neural network to classify sequences and reconstruct phylogenetic trees from sequence data. SOTA follows the SOM (Self-Organizing Map) algorithm in growing cell structures from top to bottom dynamically until a desired (user-provided) taxonomic level is reached. Recently CNNs have been used to infer the unrooted phylogenetic tree on four taxa (called a quartet)^111,112^. Authors used simulated data for training a classifier which assigns sequences to their phylogenetic tree (Fig. 3). But an analysis of the performance of the method of Zou et al.^112^ by Zaharias et al.^113^ shows that CNNs were not as accurate as other standard tree estimation methods, e.g., maximum likelihood, maximum parsimony, and neighbor joining, neither in terms of quartet estimation nor in terms of full tree estimation, especially when the sequence length was relatively short and/or rates of evolution were not sufficiently low. A potential workaround is to approach phylogeny inference as a graph generation problem, a more complex learning task.

Distance-based methods are another class of commonly used techniques for phylogenetic inference among which the neighbor joining method is the most common one, and DL has been applied to improve the distance representation. Jiang et al.^114^ addressed the phylogenetic placement problem, i.e., the problem of adding a new taxon to a given tree without having to rebuild the tree from scratch, by training a CNN using a simulated backbone tree and sequences. Given the backbone tree with its associated and query sequences, the model outputs an embedding of the query and reference species which can be used as input to some distance-based phylogenetic placement tools, which then places the query sequences onto the reference tree. Bhattacharjee et al.^115^ addressed the data imputation problem in the incomplete distance matrix using autoencoders. However, the key limitation of these methods is that trees cannot be reliably embedded into a Euclidean space of low dimensions^116^. Hyperbolic space, on the other hand, has been demonstrated to be more suitable for representing data with hierarchical latent structure^117^.

Other applications have used DL to aid in a more traditional inference pipeline. For example, the particular likelihood model to use for a maximum-likelihood search is often taken for granted as user decision, but a recent method used DL to optimize this decision^118^. In another case, DL was used to aid decision-making in the tree-search algorithm used in a traditional maximum likelihood heuristic. Finally, a very recent application uses a sparse learning model for something almost like the reverse process: given a phylogeny, it identifies the portions of a genome that most directly explain or relate to that model^119^. This can be used to validate phylogenetic inference as well as guide downstream analyses such as hypothesis generation and testing.

A traditional problem is the inference of perfect phylogeny where every site in the sequences mutates at most once along the branches of the tree. The problem of determining whether a perfect phylogeny exists and inferring it, if one exists, from binary data that is assumed to be correct is polynomially solvable. However, if the data is assumed to have errors, one approach to inferring a perfect phylogeny is by solving the minimum-flip problem: given a binary matrix of mutations - where each entry represents the presence (state 1) or absence (state 0) of mutation in a sample and a site - that does not admit a perfect phylogeny, the minimum number of “state flips" (from 0 to 1 or 1 to 0) to the data is sought so that a perfect phylogeny is admitted. Sadeqi Azer et al.^120^ used an existing DL framework originally designed for solving the traveling salesman problem to tackle this problem^121^. Here, the input consists of the inferred single-nucleotide variations (SNVs) in single cells across different sites. The output is a matrix that admits a perfect phylogeny with the minimum number of state flips from the input matrix. The input matrix is flattened and passed through convolutional layers for encoding. The encoded data is fed to a Long Short Term Memory (LSTM) layer as a decoder. Then, an attention layer takes the outputs of the LSTM layer to score the entries of the mutation matrix according to the impact that flipping them might have on minimizing the overall number of state flips. This architecture results in a probability distribution on the entries of the input matrix that is used for flipping them. The model is trained using simulated data where the matrix and the number of flips to perform are provided. The key limitation of this approach is that there is no guarantee that the output admits a perfect phylogeny because the cost function might not be fully optimized.

Taken altogether, these related successes are impressive, but given the challenges outlined above it is difficult to conceive of an end-to-end DL model to directly estimate phylogenetic trees from raw data in the near future. And if one were to be developed, given its reliance on (likely simulated) training data, its applicability to actual biological sequences will need to be carefully validated before traditional phylogenetic methods are displaced.

## General challenges for DL in the biosciences

Not all applications of DL have been equally successful in computational biology. While in some areas such as protein structure prediction and genome editing DL has found major success, in other areas like phylogenetic inference, DL has faced major hurdles (Table 1). Most common issues faced by DL approaches stem from the lack of annotated data, inherent absence of the ground truth for non-simulated datasets, severe discrepancies between training data distribution and real-world test (e.g., clinical) data distribution, potential difficulties in result benchmarking and interpretation, and finally overcoming the biases and ethical issues in datasets and models. Additionally, with the growth of the data and DL models, training efficiency has become a major bottleneck for progress.

Specifically, the success of DL in different subareas in computational biology highly relies on the availability and diversity of standardized supervised and unsupervised datasets, ML benchmarks with clear biological impact, the computational nature of the problem, and the software engineering infrastructure to train the DL models. The remaining challenges of DL in computational biology are tied with improving model explainability, extracting actionable and human-understandable insights, boosting the efficiency and limiting the training costs, and finally mitigating the growing ethical issues of DL models; innovative solutions are emerging in DL and computational biology communities (Table 2). We will now review two key areas for improvement: (i) Explainability and (ii) Training efficiency.

### Explainability

Perhaps one of the most critical limitations of DL models today, especially for biological and clinical applications, is that they are not as explainable as the simpler regression models in statistics; it is challenging to explain what each node of the network represents and how important it is to model performance. The highly nonlinear decision boundaries of DNNs and their overparameterized nature, which enable them to achieve high prediction accuracy, make them hard to explain as well. This lack of explanability becomes an important issue in computational biology, because trustworthiness of DNNs is arguably one of the most pressing problems in biological and sensitive clinical decision making applications. In fact, in biology often the question of why a model can predict well is as important as how accurately it can predict a phenomenon. For example in protein structure/function prediction we would like to know what rules in a predictive model govern the 3D geometry of a protein and its properties; in genome editing we aim to understand the biological DNA repair processes inferred from CRISPR models; in systems biology we aim to know the specific molecular differences that give rise to different phenotypes; in phylogenetics we aim to know the features that enable us to infer a phylogenetic tree. Addressing these questions are key in producing biological knowledge and creating actionable decisions in the clinical settings.

There have been some efforts in the ML community to develop methods to explain “black-box” DL models in the past few years^122^. Earlier works were developed in computer vision and biomedical applications, some of which have been applied to problems in computational biology as well. Activation maximization is a large class of algorithms which searches for an input which maximizes the model response typically by using gradient descent^123,124^; the idea is to generate an input that best symbolizes an outcome. To make them human-interpretable, the input gets regularized using closed-form density functions of the data or GANs that mimic the data distribution. Methods that address the explainability question use more direct ways to gain insights from the NN function using their Taylor expansion^125^ or Fourier transform^42,62^. The explanation takes the form of a heatmap which shows the importance of each input feature. Sensitivity analysis is another popular method of this sort which finds the input features to which the output is most sensitive to using backpropagation^126^; this has been used for classification and diagnostic prediction of cancers using DNNs and gene expression profiling as well^127^. LIME^128^ is a popular sensitivity analysis method which learns an interpretable model locally around the prediction. Simonyan et al.^124^ proposed using the gradient of the output with respect to pixels of an input image to compute a saliency map of the image. To avoid the saturation effect in perturbation-based and gradient-based approaches, DeepLIFT^129^ decomposes the output prediction of a neural network on a specific input by backpropagating the contributions of all neurons in the network to every feature of the input. SHAP^130^ unifies these approaches using a theoretically grounded method which assigns each feature an importance value for a particular prediction. Finally, GNNExplainer^131^ is a new approach among a family of methods which provide interpretable explanations for predictions of GNN-based models on graph-based DL tasks. Given an instance, GNNExplainer identifies a compact subgraph structure and a small subset of node features that have a crucial role in GNN’s prediction.

The efforts towards developing tools for explanation of DNNs are still in their infancy and are rapidly growing; challenges still abound towards a fully explainable systems in biology. The key problem is that the current general purpose methods to explain DL models are not sufficient especially in the clinical settings^132^. For the scientist and clinicians to trust these black box models they need to be able to explain themselves in a human-understandable fashion with quantifiable level of uncertainty, summarize the reasons for their behaviours, and suggest the additional steps (e.g., experiments, clinical studies, etc.) required to be able to reliably defend their decisions. We speculate that the new generation of explainable methods focus on helping these black-box models to transition from hypothesis generation machines into hypothesis testing ones which can communicate easier with medical practitioners.

### Training efficiency

Despite the high accuracy of many DL approaches, their performance often comes at a high monetary and computational cost. For example, the monetary cost of consumed power and computation time is estimated to be up to hundreds of thousands of US dollars to train a single model^133^. The extreme costs of large DL models can prevent broader research community from reproducing and improving upon the current results. Thus, it is practical to consider lower-cost alternatives that are available and feasible for researchers with more modest resources. These issues are relevant for applying DL to computational biology. For instance, training the state-of-the-art protein structure prediction model AlphaFold2 requires computational resources equivalent to 100–200 GPUs running for a few weeks^21^. In the following paragraphs, we discuss common strategies utilized by the DL community to decrease the memory and computation cost in training, and potential directions for applying similar strategies to improve the efficiency of DL models in computational biology.

The most direct method of reducing the training cost of a DL method is to perform transfer learning on the available pretrained general model, instead of training the new model from scratch. It’s a common approach in training DL models for NLP tasks, and it has been shown that general language knowledge models are a good starting point for various different NLP tasks^134^. This approach can be adopted in computational biology, if all downstream tasks can start with a general model on biological data. For example, Zaheer et al.^135^ trained a general human DNA sequence model based on human reference genome GRCh37, with self-supervised learning (masked DNA sequence prediction and next DNA sequence segment prediction). Subsequently, they have shown successful downstream task (Promoter Region Prediction) performance by solely applying transfer learning on the general model. Using pretrained models largely decreases (i) the size of task-specific datasets needed for training; and (ii) the total amount of local training needed for certain tasks that researchers are interested in. Thus creating general models that can be shared and used by the entire research community will greatly reduce the resources needed for training models on specific tasks by individual research groups. However, this approach will be less useful if the data distribution for different downstream tasks is drastically different compared with the data used by the general pretrained model. For instance, DeepVariant has limited applicability to non-human SNV calling due to the differences between diploid and haploid genomes, and nucleic acid distributions^4^. In these cases, we still need to train from scratch or spend a significant amount of resources on re-training the base model.

An alternative approach is to design DL model architectures with improved efficiency. As one of the most widely-studied architectures in DL, numerous low-cost variants of CNNs have been proposed. Some popular examples of efficient CNN architectures include the MobileNet family^136^, DenseNet^137^, EfficientNet^138^, and CSPNet^139^. Similarly, numerous efficiency-based architectural modifications have been proposed for the transformer model, many of which aim to reduce the quadratic computational complexity incurred by the self-attention mechanism^140^. Additionally, some transformer architectural variants explore the use of parameter sharing and factorization to reduce the memory cost of model training^141^. Going further, efficient architectural variants have been discovered for RNNs^142^ and graph neural networks (GNNs)^143,144^, including specialized architectures that are tuned for better efficiency within the biological domain^145^.

For computational biology applications, one approach for boosting efficiency relies on exploiting inherent sparsity and locality of biological data (e.g. focusing only on the SNV calls rather than the whole genome^146^). Researchers are also using transformers for DNA/RNA sequence modeling^135^, but transformer models have high training costs due to the expensive global attention mechanism. Prior domain expertise can be leveraged here to help prune attention neighborhoods, and subsequently improve training efficiency of the models. Finally, one can also change the model’s architecture during training to adaptively improve the training efficiency. The practice of model pruning, which removes unimportant parameters from the model, has become a popular method of deriving lightweight DL models^147^ in deployment.

As the amount of biological data keeps increasing, the size of the neural networks will increase as well, and lead to a higher total number of training iterations required for convergence. Therefore it’s natural to explore dataset reduction strategies as one of solutions to the efficiency challenge. One feasible proposal is to construct coresets of the training dataset^148^. This can be done by using clustering methods on the dataset and choosing centroids as the representatives of the dataset. Alternatively, dataset condensation can be achieved by selecting the data samples that can best approximate the effect of training the model on the whole dataset. An orthogonal way of solving the high training cost problem for DL is to distribute the training on several cheap low-end devices. This step will decrease the total training time by distributing training, and decrease the total budget by using multiple cheap devices with less computation power. In general, the major distributed training methods are data parallelism, model parallelism and hybrid parallel training. Data parallel training splits and distributes parts of the dataset to each device^149^, where model parallel training splits and distributes parts of the model to each device^150^. As all above methods are task-agnostic, they can be readily applied to DL models for computational biology.

### Concluding comments

In summary, while the success of DL in areas such as protein structure prediction is paradigm shifting, other areas such as function prediction, genome engineering, and multi-omics are also observing rapid gains in performance compared to traditional approaches. For other areas such as phylogenetics, classical computational approaches seem to have the upper hand in those areas. Additional advances specific to DL applied to challenges across the biosciences will further leverage domain-specific biological knowledge while striving for high explainability and improved efficiency.
